# Changes in NK Cells and Exhausted Th Cell Phenotype in RA Patients Treated with Janus Kinase Inhibitors: Implications for Adverse Effects

**DOI:** 10.3390/ijms26115160

**Published:** 2025-05-28

**Authors:** Juan José Fernández-Cabero, Carmen Lasa-Teja, David San Segundo, Alejandra Comins-Boo, Juan Irure-Ventura, David Walias Rivera, Jose Luis Martín-Varillas, Cristina Mata, Montserrat Santos, Elena Aurrecoechea, Ricardo Blanco, Marcos López-Hoyos

**Affiliations:** 1 Immunopathology Group, Instituto de Investigación Marqués de Valdecilla (IDIVAL), 39011 Santander, Spain; juan.fernandez@idival.org (J.J.F.-C.); carmenlasa19@gmail.com (C.L.-T.); david.sansegundo@scsalud.es (D.S.S.); alejandra.comins@scsalud.es (A.C.-B.); juan.irure@scsalud.es (J.I.-V.); rblancovela@gmail.com (R.B.); 2 Department of Rheumatology, Hospital Universitario Marqués de Valdecilla, 39008 Santander, Spain; 3 Department of Immunology, Hospital Universitario Marqués de Valdecilla, 39008 Santander, Spain; 4 Cantabria Tissue and Blood Bank, Fundación Marqués de Valdecilla, 39008 Santander, Spain; 5 Department of Rheumatology, Hospital de Laredo, 39770 Laredo, Spain; 6 Department of Rheumatology, Hospital Sierrallana, 39300 Torrelavega, Spain; 7 Department of Molecular Biology, University of Cantabria, 39005 Santander, Spain

**Keywords:** rheumatoid arthritis, JAKi, flow cytometry, NK cells, monocytes, Th cells

## Abstract

Recent concerns regarding the safety of Janus kinase inhibitors (JAKis) have prompted investigation into their impact on immune cell subsets in rheumatoid arthritis (RA) patients. This study aims to analyse alterations in immune cell populations induced by JAKis that may contribute to adverse events, such as infections or malignancies. This study included 78 RA patients meeting ACR/EULAR criteria with an established treatment with JAKis (tofacitinib, baricitinib, upadacitinib, or filgotinib), 20 healthy donors, and 20 RA patients treated with biological disease-modifying antirheumatic drugs (bDMARDs). Peripheral blood mononuclear cells (PBMCs) were immunophenotyped directly after isolation using multiparametric flow cytometry to characterise innate and adaptive immune-cell subsets. JAKi-treated patients showed a significant reduction in cytotoxic NK Dim (CD3−CD56+CD16+) cells and in the percentage of NK Dim cells expressing the activation marker Nkp30. In CD4+ T cells, the percentage of Th17 (CD3+CD4+CD45RA+CCR6+CXCR3−), Th1-17 (CD3+CD4+CD45RA+CCR6+CXCR3+), and central memory (CM, CD3+CD4+CD45RA+CD62L+) cells was lower in the JAKi group, while effector memory (EM, CD3+CD4+CD45RA−CD62L−) and terminally differentiated CD45RA (TEMRA, CD3+CD4+CD45RA+CD62L−) T helper cells were increased compared to healthy and bDMARD-treated controls. The reduction in NK Dim and Th1-17 cells and the increase in exhausted Th subsets suggest a potential compromise in antiviral immunity and balanced immune responses in JAKi-treated RA patients. These alterations may contribute to an increased risk of infections or malignancies.

## 1. Introduction

Rheumatoid arthritis (RA) is a systemic autoimmune disease characterised by both articular and extra-articular manifestations. It is commonly a chronic process that combines periods of remission with acute phases when uncontrolled inflammation occurs [1]. While the causes of RA are not fully clarified, it is understood that its origin is influenced by both genetic and environmental factors [1].

While biological disease-modifying antirheumatic drugs (bDMARDs) have been an important milestone, sustained remission is far from being achieved in most patients [2]. Besides, the use of bDMARDs may be difficult to manage when adverse events appear due to their pharmacokinetics characteristics [3,4]. Additionally, they need subcutaneous and/or intravenous administration. A new therapeutic option to fill this gap is JAK–STAT inhibitors (JAKis), which are the most recent drugs introduced in the clinical management of RA. These are a group of targeted synthetic disease-modifying antirheumatic drugs (tsDMARDs) that block the JAK–STAT signalling pathway, which is shared with other cellular functions, such as haematopoiesis, immune system balance, inflammation, tissue repairing, or apoptosis [5]. There are four different JAK proteins: JAK1, JAK2, JAK3, and TYK2, and the inhibition produced by these drugs can be selective to one JAK or non-selective. At this moment, there are four JAKis used in RA: tofacitinib (tofa); baricitinib (bari), which are non-selective; and upadacitinib (upa) and filgotinib (filgo), which are JAK1 selective [5]. A Swedish cohort reported JAKis to be slightly more efficient in pain management than tumour necrosis factor inhibitors (TNFi), a bDMARDs group of drugs, and similar to other non-TNFi bDMARDs [6].

While these drugs are very effective, they present some adverse effects, namely, opportunistic infections, such as tuberculosis and herpes zoster virus reactivation; thromboembolism; and a possible increase in the risk of neoplasia appearance [7,8]. These adverse effects may not have the same prevalence for all approved JAKis [9], which might be due to their different specificities and specific changes produced in the immune system [10,11]. We hypothesise that non-selective inhibitors may lead to greater subset changes.

T cells, specifically T helper (Th) 1 and T cytotoxic (Tc) 1 cells, and natural killer (NK) cells have an important role in the defence against intracellular threats, where the response against viruses and the appearance of malignancies have special relevance.

Different in vitro studies in healthy donors have found that JAKis can impair the proliferation capacity in T cells under different stimuli [12,13]. In addition, due to a bigger impact on cluster differentiation (CD) 8 T cells, the CD4/CD8 ratio is increased by tofacitinib [14]. A phase I study on healthy patients treated with tofacitinib reported an increase in the naïve (CD3+CD4/CD8+CCR7+CD45RA+) and central memory (CD3+CD4/CD8+CCR7+CD45RA−) helper and cytotoxic T cells, accompanied by a decrease in the effector (CD3+CD4/CD8+CCR7−CD45RA+) and effector memory (CD3+CD4/CD8+CCR7−CD45RA−) cells. The changes were reversed after withdrawal of the drug [15]. Moreover, bari [12] and tofa [16] have been demonstrated to inhibit the in vitro differentiation of B cells to plasmablasts.

The effect of JAKis on NK cells might be the cause for some adverse reactions, as previously reported [17]. The researchers carried out several in vitro experiments and found that patients treated with JAKis presented different alterations in the phenotype and function of NK cells, suggesting it is the cause of an impaired antitumour response. Those alterations consisted of a significant decrease in the surface expression of the activation marker CD69 in patients treated with tofa when compared to those treated with methotrexate and bari. When performing in vitro tests, they found alterations in the surface expression of CD57, NKp30, NKp44, NKp46, NKG2D, and CD16. In addition, they found impaired production of tumour necrosis factor (TNF) and interferon γ (IFN-γ) in tofa-treated peripheral blood mononuclear cells (PBMCs) after NKp30 stimulus and reduced expression of CD107a on NK cells exposed to tofa and co-cultured with tumour cell lines. While they open a new way of understanding the side effects of JAKis, these results need to be corroborated in real patients with in vivo studies. Moreover, biological complexity, comorbidities and their treatments, pharmacokinetic intra- and inter-patient variability, or infectious disease exposure can influence the immune system subsets through several pathways.

In this work, we aim to determine the in vivo effect of JAKis in the different subsets of the innate and adaptive immune system by comparing them with healthy and RA controls. With this study, we intend to help clarify the mechanism of action of these drugs and their relationship with the adverse events detected. The findings obtained could potentially provide monitoring strategies to anticipate and avoid their appearance.

## 2. Results

### 2.1. Clinical Characteristics of Patients Included in This Study

Seventy-eight patients with RA, according to ACR/EULAR criteria, with an established JAKi treatment were selectively and actively recruited: 36 (46.15%) were treated with bari, 19 (24.36%) with filgo, 14 (17.95%) with tofa, and 9 (11.54%) with upa. Only 24 of them (30.77%) were treated just with JAKi, 37 (47.44%) were at least also prescribed prednisone, 29 (37.18%) were treated with a cDMARD, and 12 (15.38%) were treated with both prednisone and another cDMARD. The characteristics of the patients are described in Table 1. Patients treated with the newer JAKis (filgo and upa) more frequently received a previous JAKi. Twenty RA patients from the control group were recruited: nine (45%) were being treated with abatacept and 11 (55%) with tocilizumab.

To dismiss the possible influence of the concomitant medication in the results of our analysis, we compared the populations obtained in the patients treated with prednisone with those that were not, and patients prescribed with cDMARDs compared to those that were not. These analyses did not find any statistically significant difference, so we assume that the differences found are caused by JAKis and not by the concomitant medication.

Considering the data included in the present study, 24.49% of RA patients were negative for RF and ACPA, which is consistent with previously published data, where it is indicated that the prevalence of RA patients showing negative RF/ACPA results may vary depending on the subjects’ selection, but around 20–30% of patients studied in cohorts and clinical trials present seronegative results [18]. In addition, among RA seropositive patients for RF and/or ACPA, especially in those positive for ACPA, the presence of these autoantibodies is associated with disease severity [19].

### 2.2. Effect of JAKi on the Frequencies of Circulating Adaptive Immune Cells

Among the T cells, we found statistically significant differences in the percentage of memory T helper cells (CD3+CD4+CD45RA−), which was decreased in the JAKi group in comparison to the healthy and RA controls [50.72 (39.26–61.03), 64.12 (57.14–72.71), and 64.15 (55.08–75.42) *p* = 0.008 *p* = 0.007, respectively].

On the differentiation status of the T cells, we found statistically significant differences in the percentage of effector memory cells (EM, CD3+CD45RA−CD62L+) [23.78 (14.92–30.72), 14.14 (9.95–18.07), and 13.18 (10.09–18.39), *p* < 0.001 and *p* < 0.001] between the JAKi patients, healthy controls, and RA control group and among the central memory cells (CM, CD3+CD45RA+CD62L+) [31.87 (23.60–38.95) and 38.94 (33.12–47.11), *p* = 0.008] between the JAKi patients and healthy controls.

**Figure 1:**
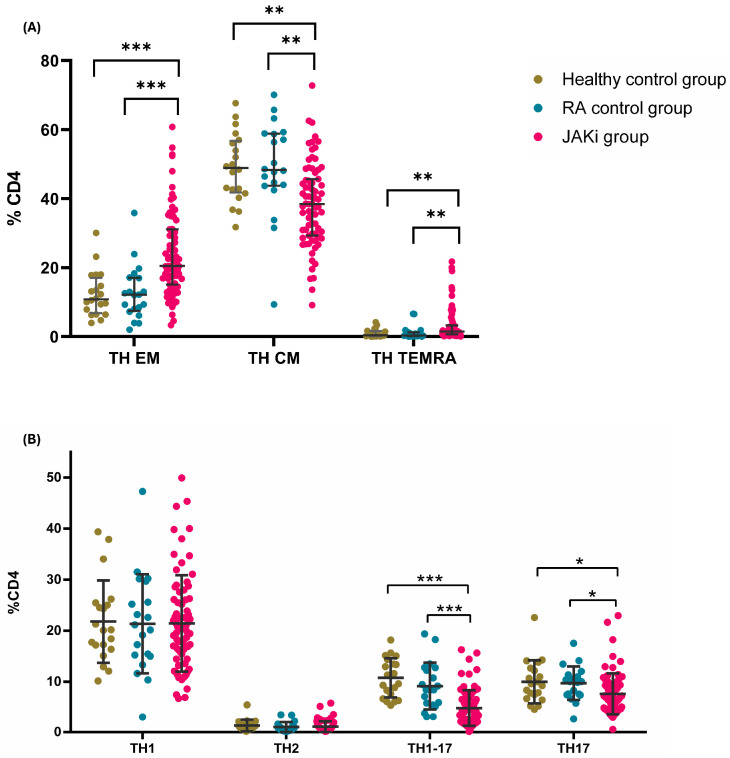
Differentiation status and percentage of T helper subsets in the three groups studied. (**A**) Dot-plots depicting the percentage of T helper according to their differentiation status, categorised as effector memory T helper (TH EM, CD3+CD4+CD62L−CD45RA−), central memory T helper (TH CM, CD3+CD4+CD62L+CD45RA−), and effector memory re-expressing RA T helper cells (TH TEMRA, CD3+CD4+CD62L−CD45RA+) among healthy (green) patients, the rheumatoid arthritis control group (blue), and the JAKi group (pink). (**B**) Dot-plots depicting the percentage of T helper subsets, categorised as T helper 1 (TH1, CD3+CD4+CD45RA−CXCR3+CCR6−), T helper 17 (TH17, CD3+CD4+CD45RA−CXCR3−CCR6+), T helper 1-17 (TH1-17, CD3+CD4+CD45RA−CXCR3+CCR6+), and T helper 2 (TH2, CD3+CD4+CD45RA−CXCR3−CCR6−CD294+) among healthy patients (green), the rheumatoid arthritis control group (blue), and the JAKi group (pink).* *p* < 0.05, ** *p* < 0.01, and *** *p* < 0.001.

These differences were also detected among CD4 positive T cells when comparing the EM and CM T helper subset in the JAKi group with healthy and RA controls [20.47 (14.77–30.69), 10.79 (7.16–16.25), and 12.12 (7.62–17.05); *p* < 0.0001 in both cases; and 38.40 (29.26–45.66), 48.87 (42.07–56.52), and 48.32 (43.82–58.8), *p* = 0.002 and *p* = 0.001, respectively]. In addition, terminally differentiated CD45RA (TEMRA, CD3+CD4+CD45RA+CD62L−) T helper cells were increased in patients treated with JAKis in comparison with healthy and RA controls [1.50 (0.66–3.13), 0.38 (0.14–1.55), and 0.33 (0.14–1.18), *p* = 0.005 and *p* = 0.003]. This is represented in Figure 1A.

When comparing the different subsets of the T helper cells, we found that the Th17 subset was decreased in the JAKi group when compared to the healthy and the RA controls [6.86 (4.66–9.44), 9.38 (7.06–12.40), and 10.01 (7.49–11.15) *p* = 0.023 *p* = 0.012, respectively]. The percentage of Th1-17 was also decreased in the group treated with JAKis in comparison to the healthy and RA controls [3.43 (2.40–6.48), 10.43 (7.10–13.88), and 8.38 (5.59–12.46) *p* < 0.0001 and *p* < 0.0001]. We found statistically significant differences in the percentage of Tc1, which was decreased in the JAKi patients in comparison to the healthy controls [22.46 (16.18–30.75) and 30.19 (24.62–36.27) *p* = 0.037], as represented in Figure 1B.

**Figure 2:**
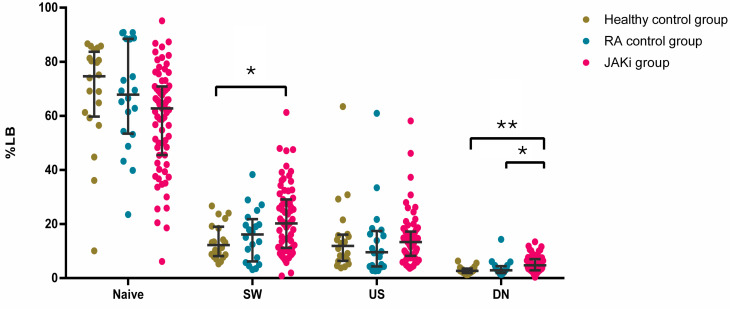
Differentiation status of B cells. Dot-plots depicting the percentage of B cells according to their differentiation status, categorised as naïve (CD19+IgD+CD27−), switched memory B cells (CD19+gD−CD27+), unswitched memory B cells (CD19+IgD+CD27+), and double-negative B cells (CD19+CD27−IgD−) among healthy (green) patients, the rheumatoid arthritis control group (blue), and the JAKi group (pink). * *p* < 0.05 and ** *p* < 0.01.

The differentiation status of B cells is represented in Figure 2. Within those cell subsets, we found statistically significant differences in the percentage of double-negative B cells between the JAKi patients and both the RA and healthy control groups [8.51 (5.72–14.09), 8.32 (3.58–10.32), and 8.91 (6.05–13.1); *p* = 0.021 and *p* = 0.002, respectively]. The percentage of switched B memory cells was increased in the JAKi patients in comparison to the healthy group [20.25 (11.18–29.08) and 12.24 (8.31–18.82), *p* = 0.018]. CD21 low B cells were increased in the JAKi patients in comparison to the RA control group [4.44 (3.05–7.97) and 3.16 (1.42–4.75), *p* = 0.045].

### 2.3. Effect of JAKi on Peripheral Blood Innate Immune Cells

**Figure 3:**
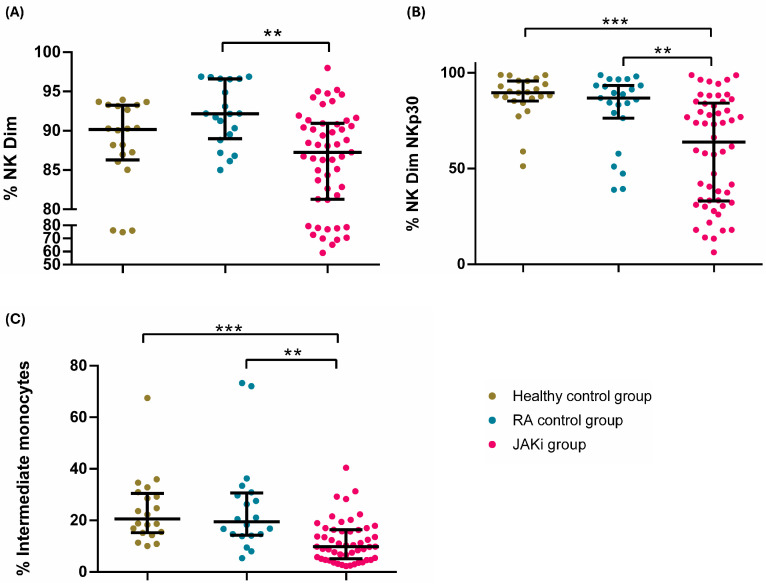
Relevant differences among the innate immune subsets. (**A**) Dot-plots depicting the percentage of NK Dim cells (CD3−CD56+CD16+) among healthy patients (green), the rheumatoid arthritis control group (blue), and the JAKi group (pink). (**B**) Dot-plots depicting the percentage of NK Dim cells expressing NKp30 among healthy patients (green), the rheumatoid arthritis control group (blue), and the JAKi group (pink). (**C**) Dot-plots depicting the percentage of intermediate monocytes (CD14+CD16+) among healthy patients (green), the rheumatoid arthritis control group (blue), and the JAKi group (pink). ** *p* < 0.01, and *** *p* < 0.001.

Among NKs, as shown in Figure 3A, a significant decline in the percentage of NK Dim, an NK subset with cytotoxic action, was measured in the JAKi group juxtaposed to the RA group treated with bDMARDs (92.18 (89.18–96.59) vs. 87.28 (81.29–90.93); *p* = 0.001).

As represented in Figure 3B, when focusing on the activation marker Nkp30, we found significant differences between the RA patients treated with JAKis and those treated with bDMARDs and healthy controls in the percentage of activated NK Dim expressing Nkp30 (59.51 (32.19–80.34), 89.74 (84.81–95.81), and 85.28 (67.08–93.16), respectively (*p* values: <0.001 and 0.007). In the NK CD16 subset, we found a decrease in the JAKi group in comparison to RA and healthy controls [13.89 (6.40–29.72), 40.90 (27.60–58.51), and 44.39 (28.11–71.94); *p* = 0.0001 in both cases].

Investigating monocytes, we found that the percentage of the intermediate subset was reduced in the JAKi category in relation with bDMARDs-treated patients and healthy controls [9.84 (5.12–16.36), 19.41 (14.39–30.30), and 20.55 (15.34–30.09), respectively; *p* values: <0.001 and 0.001]. These differences are depicted in Figure 3C.

In addition, we determined different cell ratios previously related to inflammation and autoimmune diseases, chiefly, lymphocytes/monocytes, neutrophils/lymphocytes, platelets/lymphocytes, and Th17/Treg. Considering the aforementioned ratios, the Th17/Treg ratio was solely noted as different among the three study groups: JAKi, healthy patients, and the RA control group [0.86 (0.68–1.27), 1.43 (0.97–1.83), and 1.35 (1.18–1.55), respectively; *p* = 0.003 and *p* = 0.006].

### 2.4. Differential Impact of JAKi

Among the T cell subsets, we found a significant increase in the T helper subset in the patients treated with tofa in comparison to those treated with filgo [77.88 (75.15–83.60) and 66.94 (57.85–71.24), *p* = 0.025]. Moreover, we found that the patients treated with bari had a decreased percentage of Th1-17 cells in comparison to those treated with tofa and filgo [2.77 (1.83–4.30), 5.79 (2.59–8.56), and 6.19 (2.85–9.97), *p* = 0.032 and *p* = 0.024, respectively].

### 2.5. Differential Effect of Selective and Non-Selective JAKi

We found a statistically significant decrease in the percentage of the Th cells in the patients treated with specific JAKis (filgo and upa) in comparison with patients treated with non-specific JAKis (tofa and bari) [66.94 (57.85–71.32) and 75.39 (66.64–82.28), *p* = 0.006]. In addition, the percentage of Tc cells was increased in patients treated with a specific JAKi in comparison with those treated with a non-specific JAKi [23.73 (18.27–30.66) and 16.75 (13.97–24.37), *p* = 0.022]. Comparing these same groups, we found that the specific JAKi group presents a decrease in the absolute concentration of Th1-17 cells [54.71 (31.71–84.66) and 29.63 (16.52–51.52) *p* = 0.038].

The subgroup re-analysed was formed by 36 patients treated with JAKis, specifically, 16 (44.44%) with bari, 9 (25.00%) with tofa, 8 (22.22%) with filgo, and 3 (8.33%) with upa. This second phenotype analysis, focused on NK cells, did not show any relevant difference between the patients treated with the four JAKis studied.

### 2.6. Adverse Events

During a two-year follow-up period, we detected eight respiratory infections, three of which required in-patient care, one diverticulitis, one suspected spondylodiscitis, and one skin infection.

## 3. Discussion

In this study, we thoroughly described the in vivo effects of JAKis on different circulating subsets of the innate and adaptive immune system in RA patients and compared it to healthy and biological DMARDs RA controls. To our knowledge, to date, there are no such extensive studies of the in vivo effects of JAKis but only in vitro studies or specific studies of specific immune cell subsets.

According to our data, the increase in the TEMRA helper subset may show a shift in the T helper cells towards a more exhausted phenotype, but the lack of specific exhaustion markers, such as TIGIT or KLRG1, prevents us from reaching further conclusions. The exhausted status of RA patients has been measured elsewhere [20], and it has been determined that abatacept increases the cytotoxic T cell exhausted subset (CD3+ CD8+ TIGIT+ and KLRG1+). While an augment in this cell population is associated with worse outcomes in cancer and chronic viral infections, its presence is linked to better control of autoimmune diseases [21]. The differences found in the Th1-17 and Th17 subsets may have distinct consequences: Th17 has been associated with cancer and a worse outcome in tumour progression, especially in the case of chronic viral infection [22].

The effect of JAKis on innate cells differs from the one produced by bDARMDs. The decline in the cytotoxic NK subgroup and in the activation of those may elucidate part of the adverse events produced by these drugs associated with intracellular threats, namely, viral infections or a potential increase in tumourigenesis. These results are partially consistent with previous work conducted by Meudec et al. [17], as they also observed a decrease in the in vitro expression of NKp30; however, while in their case, the biggest decrease was found in tofa and upa, we did find a decrease in the tofa group but not in the upa one, which, in our case, was higher among the JAKi group and at the level of the RA control groups. In addition, they found a significant decrease in the CD57 expression in upa and tofa patients, which, in NK cells, would mean a less mature phenotype. In our case, we measured a tendency towards an increase in this subset when comparing JAKi patients with healthy and RA controls (*p* = 0.052). These differences can be due to the fact that their analysis was made in healthy donors after a six-day in vitro exposure to the drugs, whilst our study was performed ex vivo in RA patients with an established JAKi treatment. Moreover, the lack of statistically significant differences in the subgroup re-analysed focused on NK outlines the differences between the two studies and the limitations of these comparisons.

In our study group, we observed some infections that could be categorised as adverse effects associated with these drugs but not others, such as VVZ reactivation or neoplasia appearances. This could be due to several factors. First, these events have a low incidence, and the sample size may not have been large enough to detect them. Second, these patients received the gpE recombinant herpes zoster virus vaccine shortly after the analysis, further reducing the likelihood of developing this infection.

We found a slight correlation between the Simple Disease Activity Index (SDAI) and both NK CD16 and NKT cells expressing NKp30 (rs = 0.426 and 0.454). As mentioned above, NKp30 is an activation marker whose hypothetical relationship with the disease could be logically explained. The increase in this expression can be associated with an augmentation in inflammation that can be related to the patient´s state. In addition, non-classical monocytes were also correlated with the SDAI (rs = 0.453)

The objective of this study was to characterise the impact of JAKis on RA patients. We have shown some relevant results on the Th1-17, Th17, TEMRA, EM, and CM T cells, intermediate monocytes, and NK Dim subsets. Although our data cannot demonstrate any relationship between innate and adaptive immune cell subset changes, these phenotypical changes could contribute to the appearance of the reported adverse effects produced by JAKis. If these findings are confirmed, a surveillance scheme could be used to monitor JAKi-treated patients, and, if considered by the physician, a treatment adaptation or different prevention tactics, such as vaccination, could be applied. Conversely to our expectations, we did not find many differences between the four JAKis when comparing them by their selectivity.

While this work thoroughly describes the immune system subsets in RA patients treated with JAKis, there are several limitations. Among them is the complex profiles of these patients, with different concomitant and previous treatments, especially when taking into account the limited number of participants in our study. This complexity can be a strength, too, as our study subjects are real patients, and our findings are more representative of the clinical RA patient. Complementary functional essays should be carried out to fully elucidate the impact of JAKis on innate cell populations and their relationship with harmful events in RA patients.

## 4. Materials and Methods

This was a multicentre study that enrolled patients from three hospitals Marqués de Valdecilla University Hospital (Santander, Cantabria, Spain), Laredo Hospital (Laredo, Cantabria, Spain), and Sierrallana Hospital (Torrelavega, Cantabria, Spain). For their inclusion in this study, the patients needed to meet the following criteria: positive diagnosis for RA, according to EULAR/ACR 2010 criteria [23], and being treated with a JAKi in monotherapy or in combination with DMARDS or corticosteroids. Active herpes zoster virus or other infections, malignancies, and pregnancies were exclusion criteria to enter this study. Two control study groups were included, one consisting of healthy donor (n = 20) volunteers from the Cantabria tissue and blood bank and another one of RA patients treated with biologic DMARDs (n = 20). Both groups were age- and sex-paired with the study group.

### 4.1. Analysis by Flow Cytometry

PBMCs from peripheral heparinised blood were isolated by density gradient using Ficoll Histopaque 1077 (Sigma Aldrich, St. Louis, MO, USA). These cells were stained directly after isolation with specific monoclonal antibodies to study the different immune cell subsets and to quantify the expression of different functional markers, namely, CD57, Nkp30, Slan, and HLADR, as detailed in Table 2. The PBMCs were analysed on a DX Flex cytometer from Beckman-Coulter (Brea, CA, USA) (3 lasers and the capacity to discern between 13 fluorescences). The data obtained were interpreted with the software Kaluza Analysis, version 2.2. The gating strategies for its download are available online in the Supplementary Materials (Figure S1). A subgroup of these patients was analysed for a second time during follow-up, focusing on a more complex flow cytometer NK analysis (antibodies detailed in Table 2).

The subsets of the immune system quantified through this flow cytometry analysis are summarised in Table 3.

Different humoral parameters, such as RF and ACPA, as well as total IgA, IgG, and IgM were measured in the serum from all the patients. Moreover, a routine biochemist analysis was performed, including C-reactive protein (CRP), circulating calprotectin, hepatic proteins (ALT, AST, GGT, alkaline phosphatase), cholesterol (total, HDL, LDL), triglycerides, sodium, potassium, urea, creatinine, glucose, and bilirubin.

### 4.2. Statistical Analysis

Statistical analysis was performed with SPSS version 29.0.1.0 (IBM, Armonk, NY, USA). The results were categorised into parametric and non-parametric by the Shapiro–Wilk test. The parametric results were analysed using an ANOVA test and expressed as mean ± standard deviation; for multiple comparisons, the Bonferroni correction was applied. The non-parametric results were analysed using the Kruskal–Wallis test and expressed as median with interquartile range. All the analyses were conducted using a significance threshold of *p* value < 0.05.

The percentages of the cell populations were calculated based on their most immediate precursor within the differentiation hierarchy, e.g., naïve T helper cells are expressed as the percentage of T helper cells exhibiting this characteristic.

## 5. Conclusions

In summary, our study provides a thorough in vivo characterisation of the immunological effects of JAKis in RA patients, finding differences in both adaptive and innate immune cell subsets compared to healthy controls and RA patients treated with bDMARDs. These findings suggest that JAKi treatment may influence immune cell phenotypes towards less activated and more exhausted states, particularly in the cytotoxic NK and T cells. While these phenotypic changes may cause some of the adverse effects observed in clinical practice, such as infections, further research is needed to confirm their relationship, clinical relevance, and mechanistic basis. Although the lack of significant differences among individual JAKis and regarding JAKi selectivity limits our conclusions, these results suggest the need for further functional studies. Ultimately, a better understanding of these immunological changes could help create monitoring and preventive strategies in RA patients undergoing JAKi therapy.

## Figures and Tables

**Table 1:** Clinical characteristics of patients included in this study. ^#^ refers to patients receiving only JAKi or bDMARD as DMARDs; those patients may be treated with corticoids or NSAIDs. * Circulating calprotectin was measured afterwards in those patients with available frozen serum samples. *n* baricitinib: 31, *n* filgotinib: 17, *n* tofacitinib: 0, and *n* Upadacitinib: 6. CVRF: cardiovascular risk factors, RF: rheumatoid factor, ACPA: anti-citrullinated protein antibodies, NA: not available, NSAIDs: non-steroidal anti-inflammatory drugs, MTX: methotrexate, bDMARD: biologic disease-modifying antirheumatic drug, cDMARDS: conventional disease-modifying antirheumatic drug, AntiTNF: anti-tumour necrosis factor, Anti-IL6: anti-interleukin 6, DAS28, CRP: C-reactive protein, RAPID3: routine assessment of patient index data 3, CDAI: clinical disease activity index, ESR: erythrocyte sedimentation rate.

	Treatment or Group						
	BARICITINIB *n* = 36	FILGOTINIB *n* = 19	TOFACITINIB *n* = 14	UPADACITINIB *n* = 9	ABATACEPT *n* = 9	TOCILIZUMAB *n* = 11	Healthy Controls *n* = 20
Age, years, mean ± SD	64.4 ± 10.4	61 ± 11.4	55.5 ± 8.6	63.1 ± 7.8	66.2 ± 9	60.3 ± 10.2	60 ± 4.3
Women, n (%)	28 (77.8)	18 (94.7)	14 (100)	9 (100)	5 (55.6)	8 (72.7)	17 (85)
RA duration, months, mean ± SD	178.3 ± 135.4	256 ± 307.1	166.9 ± 65.4	65.1 ± 53.9	236.1 ± 135.9	238.1 ± 96.1	NA
CVRF, n (%)	31 (86.1)	14 (73.7)	9 (64.3)	5 (55.6)	9 (100)	9 (81.8)	NA
RF/ACPA, n (%)	25 (69.4)	17 (89.5)	10 (71.4)	6 (66.7)	8 (88.9)	8 (72.7)	1 (5)
Extra-articular manifestations, n (%)	6 (16.6)	0	2 (14.3)	0	2 (22.2)	0	NA
Erosive, n (%)	12 (33.3)	7 (36.8)	6 (42.9)	6 (66.7)	7 (77.8)	6 (54.5)	NA
Comorbidities, n (%)	34 (94.4)	18 (94.7)	14 (100)	9 (100)	9 (100)	10 (90.9)	NA
**Current treatment**							
Prednisone dose, mg/day, median [IQR]	2.5 [0–5]	2.5 [0–5]	2.5 [0–7.5]	5 [0–10]	5 [5–7.5]	0 [0–2]	0 [0–0]
NSAIDs, n (%)	12 (33.3)	6 (31.6)	9 (64.3)	8 (88.9)	6 (66.7)	8 (72.7)	NA
MTX, n (%)	7 (19.4)	4 (21.1)	4 (28.6)	1 (11.1)	1 (11.1)	2 (18.2)	0
JAKi or bDMARD monotherapy ^#^, n (%)	19 (52.8)	9 (47.4)	8 (57.1)	5 (55.5)	3 (33.3)	6 (54.5)	0
**Previous treatment**							
MTX, n (%)	36 (100)	19 (100)	14 (100)	9 (100)	8 (88.9)	10 (90.9)	NA
Other cDMARDS, n (%)	29 (80.6)	17 (89.5)	8 (57.1)	8 (88.9)	3 (33.3)	3 (27.3)	NA
AntiTNF, n (%)	29 (80.6)	16 (84.2)	13 (92.9)	6 (66.7)	3 (33.3)	5 (45.5)	NA
Anti-IL6, n (%)	15 (41.7)	14 (73.7)	7 (50)	6 (66.7)	4 (44.4)	0	NA
Previous cDMARDS, number, median [IQR]	2 [2–3]	3 [2–3]	1.5 [1–4]	2.5 [1.3–3.8]	1 [1–1]	1 [1–1]	NA
Previous bDMARDS, number, median [IQR]	2 [1–3]	2 [1.3–3]	2 [1–2]	2 [0.5–3.75]	1 [1–2]	0 [0–2]	NA
**RA status**							
DAS28, RCP, remission, n (%)	14 (38.9)	12 (63.2)	7 (50)	2 (25)	NA	NA	NA
DAS28, RCP, low disease activity, n (%)	9 (25)	0	3 (27.3)	0	NA	NA	NA
RAPID3, remission, n (%)	17 (47.2)	12/18 (66.7)	3/5 (60)	1/4 (25)	NA	NA	NA
CDAI, remission, n (%)	12 (33.3)	6 (31.6)	0	2 (33.3)	NA	NA	NA
Ultrasonography remission, n (%)	22 (61.1)	6 (31.6)	8 (57.1)	3 (50)	NA	NA	NA
**Inflammatory markers**							
CRP (mg/dL), median [IQR]	0.4 [0.4–0.4]	0.4 [0.4–0.7]	0.4 [0.4–0.9]	1 [0.4–1.4]	0.4 [0.4–0.6]	0.4 [0.4–0.4]	0.4 [0.4–0.4]
ESR (mm/1st hour], mean ± DS	15.2 ± 21.9	10.7 ± 22.8	28.4 ± 15.1	25.8 ± 27.1	25.2 ± 20.7	7.0 ± 6.7	NA
Circulating calprotectin (μg/mL), median [IQR] *	1.28 [0.98–1.8]	0.87 [0.72–1.23]	-	1.14 [0.91–1.25]	1.8 [1.01–1.85]	0.87 [0.73–1.2]	1.39 [1.01–1.65]

**Table 2:** Monoclonal antibody panel and fluorochromes for the phenotype analysis. NK: natural killer, AF: Alexa fluor, PE: phycoerythrin, ECD: phycoerythrin–Texas Red, PC5.5: phycoerythrin-cyanine 5.5, APC: allophycocyanin, APCCy7: allophycocyanin cyanine 7, PB: Pacific blue, FITC: fluorescein isothiocyanate, VB: VioBlue, BV: brilliant violet, PerCP Cy5.5: peridinin chlorophyll protein–cyanine 5.5, KrO: Krome orange.

	FL1	FL2	FL3	FL4	FL5	FL6	FL7	FL8	FL9	FL10	FL11	FL12	FL13
**T cells**	CXCR3 AF488	CD127 PE	CD62L ECD	CD4 PC5.5	CD8 PC7	CD3 APC	CD45RA AF700	CD294 APCCy7	CCR6 PB				CD25 BV785
**Monocytes and NK**	CD57 FITC	CD14 PE	CD56 ECD		NKp30 PC7	CD3 APC		CD16 APCCy7	SLAN VB			HLA-DR BV650	
**B cells**	IgD FITC				CD27 PC7	CD20 APC	CD21 APC700	CD38 APC750	CD19 PB				
**Extended NK cell analysis**	CD57 FITC	CD16 PE	CD56 ECD	LILRB (ILT2) PerCP Cy5.5		NKG2C APC	CD14 APC700	CD69 APCCy7	NKG2A BV421	CD3 KrO	NKp30BBV605	HLA-DR BV650	NKp46 BV785

**Table 3:** Cell subsets and their surface expression markers used in this study.

Cell Subset	Surface Expression Marker
T cells	CD3+
T helper cells	CD3+CD4+
T cytotoxic cells	CD3+CD8+
T helper 1	CD3+CD4+CD45RA−CXCR3+CCR6−
T helper 17	CD3+CD4+CD45RA−CXCR3−CCR6+
T helper 1-17	CD3+CD4+CD45RA−CXCR3+CCR6+
T helper 2	CD3+CD4+CD45RA−CXCR3−CCR6−CD294+
T cytotoxic 1	CD3+CD8+CD45RA−CXCR3+CCR6−
T cytotoxic 17	CD3+CD8+CD45RA−CXCR3−CCR6+
T cytotoxic 1-17	CD3+CD8+CD45RA−CXCR3+CCR6+
T cytotoxic 2	CD3+CD8+CD45RA−CXCR3−CCR6−CD294+
Effector memory T cells	CD3+CD62L−CD45RA−
Central memory T cells	CD3+CD62L+CD45RA−
Effector memory re-expressing RA	CD3+CD62L−CD45RA+
Naïve T cells	CD3+CD62L+CD45RA+
Regulatory T cells	CD3+CD4+CD127−CD25+
Effector memory T helper cells	CD3+CD4+CD62L−CD45RA−
Central memory T helper cells	CD3+CD4+CD62L+CD45RA−
Effector memory re-expressing RA T helper cells	CD3+CD4+CD62L−CD45RA+
Naïve T helper cells	CD3+CD4+CD62L+CD45RA+
Effector memory T cytotoxic cells	CD3+CD8+CD62L−CD45RA−
Central memory T cytotoxic cells	CD3+CD8+CD62L+CD45RA−
Effector memory re-expressing RA T cytotoxic cells	CD3+CD8+CD62L−CD45RA+
Naïve T cytotoxic cells	CD3+CD8+CD62L+CD45RA+
B cells	CD19+
Double negative B cells	CD19+CD27−IgD−
Naïve B cells	CD19+IgD+CD27−
Switched memory B cells	CD19+IgD−CD27+
Unswitched memory B cells	CD19+IgD+CD27+
CD21 low B cells	CD19+CD27−CD21−
Plasmablasts	CD19+CD20−CD19+CD38++CD27++
Natural killers	CD3−CD56+/CD16+
NK bright	CD3−CD56++CD16−
NK Dim	CD3−CD56+CD16+
NK CD16	CD3−CD56−CD16+
NKT	CD3+CD56+
**Monocytes**	
Classical monocytes	CD14+CD16−
Intermediate monocytes	CD14+CD16+
Non-classical monocytes	CD14−CD16+

## Data Availability

The data underlying this article will be shared on reasonable request to the corresponding author.

## Supplementary Materials

The following supporting information can be downloaded at: https://www.mdpi.com/article/10.3390/ijms26115160/s1.

## Abbreviations

The following abbreviations are used in this manuscript:

aCarp	anti-carbamylated protein antibodies
ACPA	anti-citrullinated protein antibodies
AMPA	anti-modified protein antibodies
aPAD	anti-peptidylarginine deiminases
Bari	baricitinib
bDMARD	biological disease-modifying antirheumatic drug
CD	cluster differentiation
CDAI	clinical disease activity index
cDMARD	conventional disease modifying antirheumatic drug
CEIM	Cantabria Ethical Committee
CM	central memory
CRP	C-reactive protein
CVRF	cardiovascular risk factor
DAS28	disease activity score
DMARD	Disease-modifying antirheumatic drug
EM	effector memory
ESR	erythrocyte sedimentation rate
Filgo	filgotinib
IFN-γ	interferon γ
Ig	immunoglobulin
IQR	interquartile range
JAKi	Jak–Stat inhibitors
Jak–Stat	Janus kinase–signal transducer and activator of transcription
MTX	methotrexate
NK	natural killer
NSAIDs	non-steroid anti-inflammatory drugs
PBMCs	peripheral blood mononuclear cells
RA	rheumatoid arthritis
RAPID3	routine assessment of patient index data 3
RF	rheumatoid factor
SE	shared epitopes
TC	T cytotoxic
TEMRA	terminally differentiated CD45RA
TH	T helper
TNF	tumour necrosis factor
Tofa	tofacitinib
Treg	T regulatory
tsDMARD	targeted synthetic disease-modifying antirheumatic drug
Upa	upadacitinib
