# Supplementary material for: Changes in NK Cells and Exhausted Th Cell Phenotype in RA Patients Treated with Janus Kinase Inhibitors: Implications for Adverse Effects
Source: Int J Mol Sci. 2025 May 28;26(11):5160. doi: 10.3390/ijms26115160 (PMC12155405; doi:10.3390/ijms26115160)
Supplement: Supplementary file 1 [file ijms-26-05160-s001.zip › ijms-3584672-supplementary.pptx]

## Slide 1
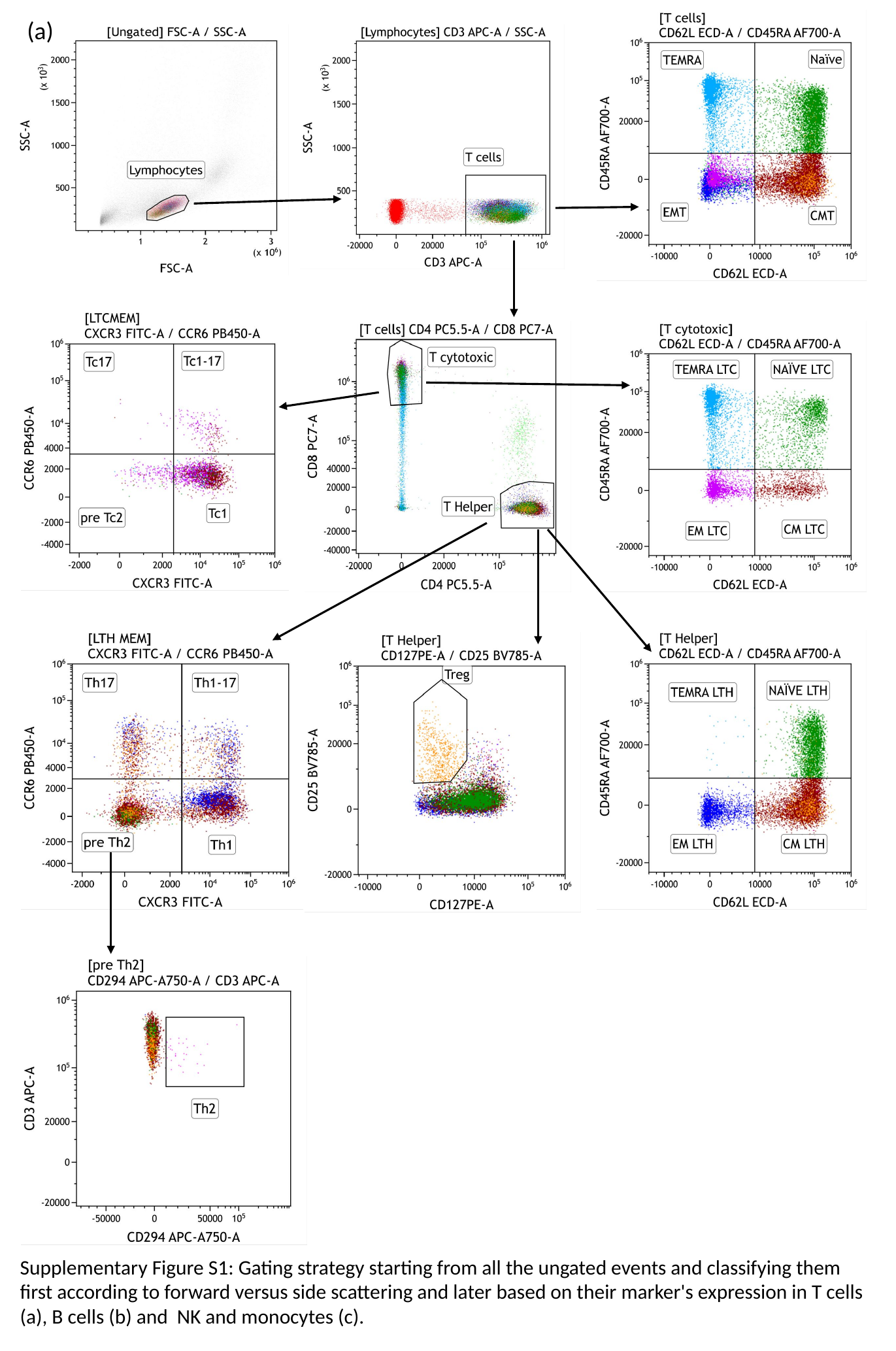

(a)
Supplementary Figure S1: Gating strategy starting from all the ungated events and classifying them first according to forward versus side scattering and later based on their marker's expression in T cells (a), B cells (b) and NK and monocytes (c).

## Slide 2
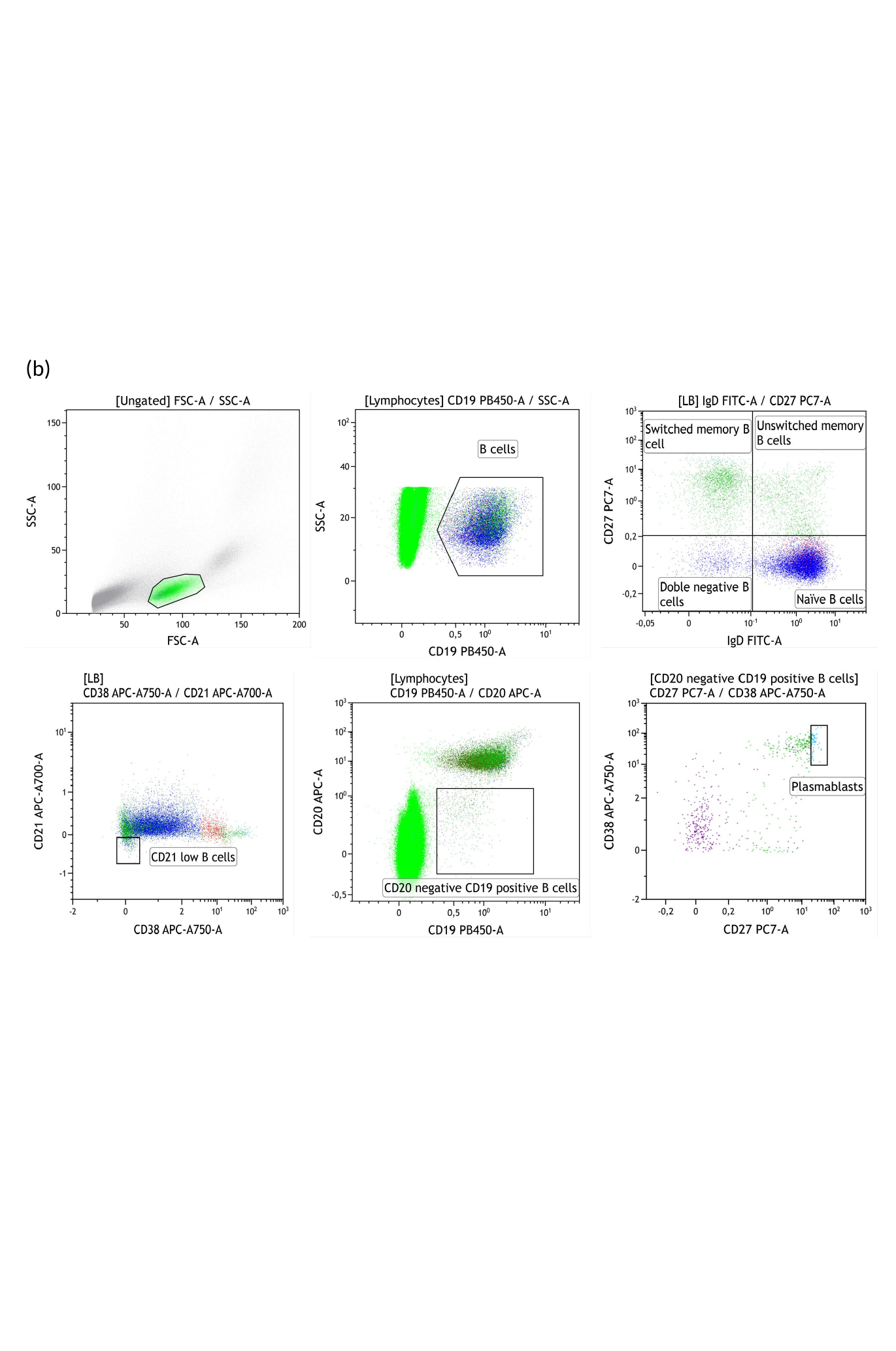

(b)

## Slide 3
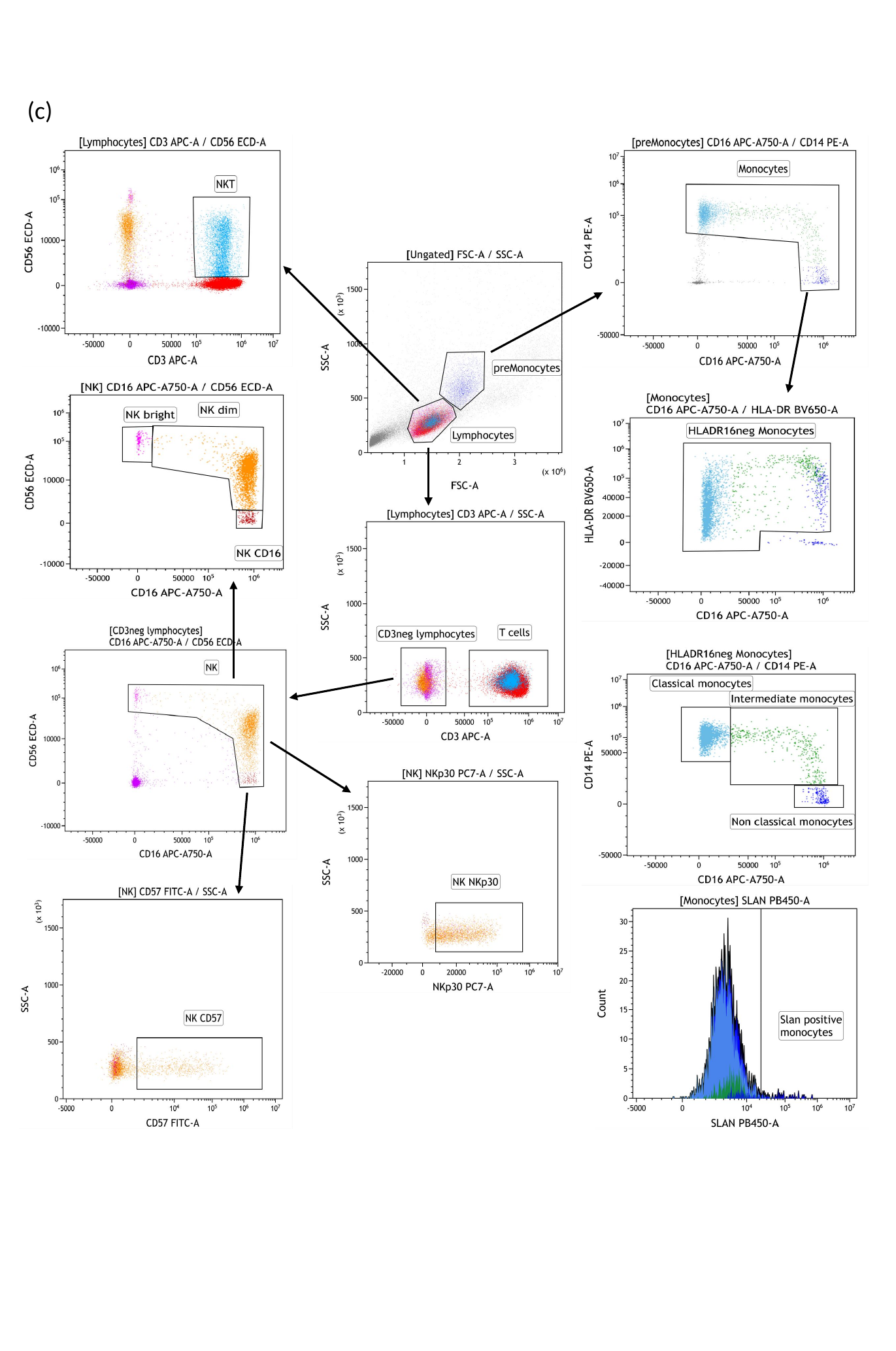

(c)
